# Nocturnal melatonin secretion and risk of type 2 diabetes: a prospective cohort study

**DOI:** 10.1210/clinem/dgaf667

**Published:** 2025-12-13

**Authors:** Einar Sojakka Smith, Anna Franzén, Mats Pihlsgård, Peter M Nilsson, Simon Timpka, Sofia Enhörning

**Affiliations:** Perinatal and Cardiovascular Epidemiology, Department of Clinical Sciences in Malmö, Lund University, Malmö 214 28, Sweden; Department of Internal Medicine, Skåne University Hospital, Malmö 214 28, Sweden; Perinatal and Cardiovascular Epidemiology, Department of Clinical Sciences in Malmö, Lund University, Malmö 214 28, Sweden; Perinatal and Cardiovascular Epidemiology, Department of Clinical Sciences in Malmö, Lund University, Malmö 214 28, Sweden; Department of Clinical Sciences in Malmö, Lund University, Malmö 214 28, Sweden; Perinatal and Cardiovascular Epidemiology, Department of Clinical Sciences in Malmö, Lund University, Malmö 214 28, Sweden; Departments of Obstetrics and Gynecology, Skåne University Hospital, Lund and Malmö 214 28, Sweden; Perinatal and Cardiovascular Epidemiology, Department of Clinical Sciences in Malmö, Lund University, Malmö 214 28, Sweden; Department of Internal Medicine, Skåne University Hospital, Malmö 214 28, Sweden

**Keywords:** melatonin, diabetes mellitus, type 2, circadian rhythm, cohort studies, biomarkers, 6-sulfatoxymelatonin

## Abstract

**Context:**

Melatonin regulates circadian rhythms and influences glucose metabolism. Altered melatonin secretion may contribute to the pathogenesis of type 2 diabetes (T2D), but prospective population-based evidence is scarce.

**Objective:**

To examine whether low nocturnal melatonin secretion is associated with an increased risk of incident T2D in adults.

**Methods:**

This prospective cohort study, with follow-up from 2013 to 2023 (median 6.5 years), included a total of 4491 adults (52% women, aged 18-75 years) without T2D and melatonin supplementation at baseline, from the Malmö Offspring Study, a population-based cohort in southern Sweden. Incident T2D was identified via national and regional health registers. Nocturnal melatonin secretion was assessed as the urinary 6-sulfatoxymelatonin-to-creatinine ratio (aMT6s/Cr) from first-morning urine samples, categorized into sex-specific quintiles.

**Results:**

During follow-up, 171 participants developed T2D. Participants in the lowest quintile of aMT6s/Cr had a higher T2D risk than those in quintiles 2 to 5 (multivariable adjusted hazard ratio [HR] 1.51, 95% CI 1.09-2.09). The association remained significant after additional adjustment for sleep duration and disruption (HR 1.54, 95% CI 1.11-2.13). When analyzing T2D development per 1-SD higher sex-standardized log aMT6s/Cr, the HR was 0.84 (95% CI 0.73-0.97). Associations were consistent across sex, age, and BMI subgroups.

**Conclusion:**

Low nocturnal melatonin secretion was independently associated with a higher incidence of T2D in adults. A key limitation is the reliance on a single morning urine sample to estimate melatonin secretion. The findings support circadian regulation as a determinant of metabolic health and warrant further investigation of melatonin pathways in diabetes prevention.

Circadian disruption has emerged as an important determinant of metabolic disease, but the role of melatonin secretion in the development of type 2 diabetes (T2D) remains uncertain (1). Melatonin is a circadian hormone that synchronizes physiological processes, including metabolic rhythms (1). Disturbance of the sleep-wake diurnal cycle, including melatonin signaling, has been linked to metabolic disturbance and elevated T2D risk (2, 3). Melatonin has an inhibitory effect on insulin release from pancreatic β-cells (4), and genome-wide association studies have identified common genetic variants in the *MTNR1B* gene, encoding the melatonin receptor type 2 (MT2), as risk factors for T2D (4-7). Among these, the most established single nucleotide polymorphism (rs10830963) is associated with elevated fasting glucose and impaired insulin secretion due to increased MT2 expression in pancreatic beta cells, amplifying melatonin's inhibitory effect on insulin release (8-11).

Although genetic variants that amplify melatonin signaling point to a higher risk of T2D, epidemiological studies quantifying melatonin secretion report the opposite pattern. Patients with established T2D often exhibit lower nocturnal melatonin concentrations than healthy individuals (12, 13). Furthermore, in a nested case-control study in older women, low nocturnal melatonin secretion was associated with higher risk of incident T2D, independent of conventional risk factors (14). Such discrepancies may reflect melatonin's context-dependent effects which vary by genetic background and timing of exposure (1, 10).

Additionally, rare loss-of-function variants in the *MTNR1B* gene have been associated with increased T2D risk, further underscoring the complexity of melatonin's role in glucose metabolism (1, 15). Given the widespread use of melatonin supplements and the therapeutic potential of intervening on melatonin receptors, disentangling when the metabolic effect of melatonin is protective vs detrimental, is of considerable clinical importance (16). However, the association between nocturnal melatonin secretion and T2D risk has not been examined in mixed-sex, population-based cohorts including younger adults.

To address this knowledge gap, we aimed to investigate whether low nocturnal melatonin secretion, measured as urinary 6-sulfatoxymelatonin-to-creatinine ratio (aMT6s/Cr), was prospectively associated with incident T2D in a large prospective cohort of adults.

## Methods

In this prospective cohort study, participants within the Malmö Offspring Study (MOS) were included. MOS is a Swedish, family-based cohort initiated in 2013 with detailed follow-up via national registers (17) and the participants in MOS are adult children and grandchildren of those enrolled in the Malmö Diet and Cancer Study Cardiovascular Cohort (17, 18). Eligible participants were 18 years or older, and all lived in the county of Skåne in southern Sweden. All participants provided written informed consent, and the study was ethically approved by the Regional Ethics committee in Lund (Dnr 2012/594 and Dnr 2020/04422 with addition 2022/06493/02).

### Study population

Participants were initially recruited from 2013 to 2021 to form the MOS cohort of 5288 participants. Exclusions were applied sequentially to remove those without any relevant baseline measurements (n = 234), blood analyses (n = 43), or urinary analyses (n = 253). Further exclusions were made for the use of melatonin supplementation (n = 1), overly diluted urine samples (defined as urinary creatinine < 0.1 mmol/L or 6-sulfatoxymelatonin below 1 ng/mL; n = 20) and participants without follow-up data on diabetes diagnosis (n = 3). Finally, individuals diagnosed with type 1 diabetes at baseline or during follow-up (n = 18) were excluded from the analyses. These steps yielded a sample of 4716 individuals with baseline data.

Participants with prevalent T2D (n = 225) were subsequently excluded from prospective analyses, yielding a sample of 4491 individuals for main analysis.

### Exposure assessment

Overnight first-morning urine samples were collected by participants at home. Urine samples were analyzed for creatinine at the Department of Clinical Chemistry, Malmö University Hospital, aliquoted, and stored at −80 °C at the Region Skåne Biobank in Lund. As an established estimate of nocturnal melatonin secretion, 6-sulfatoxymelatonin (aMT6s), the primary metabolite of melatonin (19), was measured from stored aliquots using a commercial enzyme-linked immunosorbent assay (cat. no. RE54031) from IBL-Hamburg (Hamburg, Germany). The assay had a reported limit of detection of 1.0 ng/mL, with intra-assay coefficients of variation ranging from 5.2% to 12.2% and inter-assay coefficients from 5.1% to 14.9%, according to the manufacturer. Urinary aMT6s was normalized to urinary creatinine (aMT6s/Cr) to account for sample dilution (19, 20).

### Outcome assessment

Prevalent T2D was defined as fasting glucose higher than 7.0 mmol/L at baseline or having a T2D diagnosis or getting the T2D diagnosis within 2 months after the baseline visit. Subsequent (incident) T2D was ascertained by deterministic linkage of each participant's unique personal identity number to 6 complementary nationwide or regional data sources: the Malmö HbA1c Register, the Swedish National Diabetes Register, the regional Diabetes 2000 Register for Skåne, the Swedish National Patient Register, the Cause-of-Death Register, and the Prescribed Drug Register, following the procedure described by Enhörning et al (21). The date of the last follow-up was December 31, 2023.

### Covariables

Baseline questionnaires were completed by participants to collect information on sleep quality, education level, alcohol use, physical activity, medication use, and smoking status (17). Use of β-blockers (metoprolol, bisoprolol, propranolol, carvedilol, or atenolol) and melatonin (ATC code N05CH01) was self-reported at baseline. Body mass index (BMI) was calculated from measured height in meters and weight in kilograms as kg/m^2^. Resting blood pressure was measured as the mean of 2 readings in the supine position after 10 minutes rest by using an automatic device (Omron). Overnight fasting plasma was analyzed for lipids, glucose, and creatinine at the Department of Clinical Chemistry, Skåne University Hospital, Malmö.

### Statistical analysis

Differences in nocturnal melatonin secretion between participants with and without prevalent T2D were assessed cross-sectionally using a Student *t* test on aMT6s/Cr values, which were log-transformed to reduce skewness and approximate normality, then standardized using sex-specific z-score.

For prospective analyses, individuals with prevalent T2D were excluded, and aMT6s/Cr was ranked within each sex to establish sex-specific quintile cut-points. The resulting 5-level categorical variable, merging quintile indicators from both sexes, was used to describe baseline characteristics: continuous covariates were compared across quintiles with one-way analysis of variance (ANOVA), whereas categorical covariates were cross-tabulated by quintile and evaluated with χ² tests. In parallel, the log-transformed aMT6s/Cr was also standardized (z-scored) separately for men and women to be used as a continuous predictor in secondary analyses.

To investigate the crude association between aMT6s/Cr quintiles and T2D incidence, we used Kaplan-Meier analysis, with main analyses comparing the lowest quintile (Q1) with quintiles 2 to 5 (Q2-Q5). This grouping was chosen to capture individuals with markedly low secretion while maintaining statistical power and interpretability. In addition, Kaplan-Meier curves were constructed for all quintiles, and Q1 vs Q2-Q5 was further stratified by sex.

To assess hazard ratios for T2D by aMT6s/Cr quintiles (Q1 vs Q2-Q5), we used Cox proportional hazards regression models. In Model 1, adjustment for age was included. In Model 2, additional adjustment for BMI was included. In Model 3, further inclusion of systolic blood pressure, triglycerides, low-density lipoprotein (LDL)-cholesterol, high-density lipoprotein (HDL)-cholesterol, glucose, plasma creatinine, education level, smoking status, physical activity, and β-blocker usage was performed. Finally, Model 4 additionally adjusted for self-reported sleep disruption and sleep duration. Because sleep may function as both a mediator and a proxy confounder, we present models both with and without adjustment for sleep characteristics.

For additional analyses, Cox models were constructed using the same adjustment models, but with log-transformed aMT6s/Cr standardized by sex using z-score as a continuous exposure to evaluate robustness and comparability with prior studies. To evaluate whether the association between aMT6s/Cr and T2D differed by sex, age, or BMI, we included interaction terms for both the quintile-based and continuous aMT6s/Cr exposures, respectively. Proportional hazards assumptions were evaluated using Schoenfeld residuals.

To address missing covariable data (sleep duration, sleep disruption, alcohol intake, smoking status, education level, physical activity, and β-blocker usage, 7%-11% missing), multiple imputation by chained equations (m = 20) was applied using the mice package in R (22). The imputation model included aMT6s/Cr, all covariables from Model 4, the event indicator (incident T2D), and the log-transformed Nelson–Aalen cumulative hazard (interpolated to each participant's follow-up time) to represent survival time, as recommended for time-to-event analyses (23). Prespecified interaction terms (aMT6s/Cr × sex, aMT6s/Cr × age, and aMT6s/Cr × BMI) were specified as passive to maintain algebraic consistency across imputations. Cox models were fitted within each imputed dataset and combined using Rubin's rules (23). To address missing data on melatonin supplementation (n = 320), a sensitivity analysis excluding individuals with missing data on melatonin supplementation from the Cox regression analyses was conducted.

All analyses were conducted in R version 4.4.2 using packages “survival” for survival analyses and “mice” for multiple imputations (22, 24).

## Results

**Figure 1:**
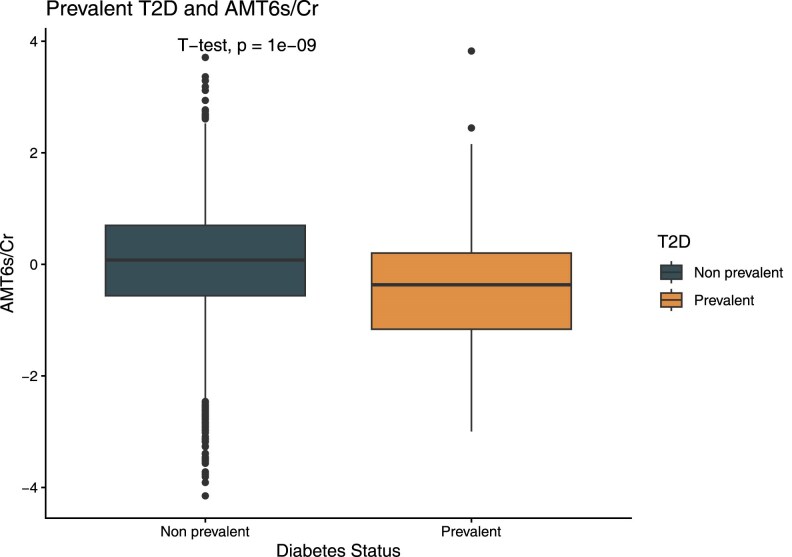
Distribution of log-transformed, sex-standardized urinary 6-sulfatoxymelatonin-to-creatinine ratio (aMT6s/Cr) by prevalent type 2 diabetes (T2D) status at baseline. Boxes represent the interquartile range, and whiskers denote 5th to 95th percentiles. Comparison was made by 2-sided Student *t* test. Abbreviations: aMT6s/Cr, urinary 6-sulfatoxymelatonin-to-creatinine ratio; T2D, type 2 diabetes.

Among 4716 participants with a mean age of 43 years (range, 18-75 years), 225 had prevalent T2D (Table 1). Individuals with prevalent T2D at baseline had lower median aMT6s/Cr (23.7 ng/mg, 5%-95% range 6.7-77.0 ng/mg) compared to individuals without T2D (35.6 ng/mg, 5%-95% range 9.9-103.5 ng/mg) (Student *t* test *P* < .001 comparing the log-transformed melatonin ratio, Fig. 1). For subsequent analyses, individuals with prevalent T2D were excluded as described below, resulting in a dataset of 4491 participants with no prevalent diabetes.

**Table 1:** Baseline characteristics of the Malmö Offspring Study cohort

Characteristics	Participants (N = 4716)
Age, y	42.6 (14.6)
Sex	
Men	2286 (48%)
Women	2430 (52%)
aMT6s/Cr, ng/mg	43.2 (34.8)
BMI, kg/m²	26.2 (4.8)
Systolic BP, mmHg	116.1 (15.5)
Glucose, mmol/L	5.4 (1.0)
Triglycerides, mmol/L	1.2 (1.0)
HDL-C, mmol/L	1.6 (0.5)
LDL-C, mmol/L	3.2 (1.0)
Plasma creatinine, µmol/L	77.4 (15.7)
Prevalent T2D	
No prevalent T2D	4491 (95%)
Prevalent T2D	225 (5%)
Current smoker (7% missing)	677 (14%)
Sleep duration, h (11% missing)	6.9 (1.0)
β-blocker use (7% missing)	191 (4.4%)

Continuous variables are expressed as mean (SD); categorical variables as n (%).

Abbreviations: aMT6s/Cr, urinary 6-sulfatoxymelatonin-to-creatinine ratio; BMI, body mass index; BP, blood pressure; HDL-C, high-density lipoprotein cholesterol; LDL-C, low-density lipoprotein cholesterol; T2D, type 2 diabetes.

In participants without T2D at baseline, the median aMT6s/Cr was higher in women (44.3 ng/mg) than in men (28.1 ng/mg).

Higher aMT6s/Cr was also associated with younger age, lower BMI, and a more favorable cardiometabolic profile characterized by lower LDL-cholesterol, higher HDL-cholesterol, reduced triglyceride concentrations, lower fasting glucose, lower serum creatinine, and lower systolic blood pressure (Table 2).

**Table 2:** Participant characteristics across sex-specific quintiles of urinary 6-sulfatoxymelatonin-to-creatinine ratio (aMT6s/Cr)

	aMT6s/Cr quintiles					
	Q1	Q2	Q3	Q4	Q5	*P* value
aMT6s/Cr, ng/mg	14.0 (5.5)	26.1 (6.3)	36.7 (8.7)	51.1 (12.8)	90.7 (44.7)	<.001
Age, y	45.9 (14.2)	43.2 (14.1)	40.9 (14.1)	41.0 (14.4)	39.1 (14.4)	<.001
BMI, kg/m²	27.2 (5.1)	26.6 (4.7)	26.0 (4.0)	25.6 (4.4)	24.8 (4.0)	<.001
LDL-C, mmol/L	3.2 (1.0)	3.2 (1.0)	3.1 (1.0)	3.1 (1.0)	3.1 (0.9)	<.001
HDL-C, mmol/L	1.6 (0.5)	1.6 (0.5)	1.6 (0.5)	1.7 (0.5)	1.7 (0.5)	<.001
Triglycerides, mmol/L	1.2 (0.7)	1.2 (0.7)	1.1 (0.6)	1.1 (0.5)	1.0 (0.6)	<.001
Glucose, mmol/L	5.3 (0.6)	5.3 (0.6)	5.2 (0.6)	5.2 (0.6)	5.2 (0.6)	<.001
Plasma creatinine, µmol/L	78.8 (14.3)	78.2 (14.1)	77.7 (14.0)	77.0 (19.1)	75.6 (15.5)	<.001
Systolic BP, mmHg	118.4 (15.8)	116.3 (15.1)	115.4 (14.6)	114.5 (14.6)	113.6 (15.1)	.002
Current smokers	133 (16%)	138 (17%)	117 (14%)	136 (16%)	114 (14%)	.001
β-blocker use	54 (6.5%)	31 (3.7%)	25 (3%)	20 (2.4%)	21 (2.5%)	<.001

Values are reported as mean (SD) or No. (%). *P* values are from analysis of variance for continuous variables and χ² tests for categorical variables.

Abbreviations: aMT6s/Cr, urinary 6-sulfatoxymelatonin-to-creatinine ratio; BP, blood pressure; HDL-C, high-density lipoprotein cholesterol; LDL-C, low-density lipoprotein cholesterol.

The distributions of sex, sleep duration and disruption, and alcohol-consumption frequency were comparable across sex-specific AMTs/Cr quintiles (quintile 1 range 1.9-24.8 ng/mg) (Table 3). By contrast, successively higher quintiles showed progressively lower smoking prevalence, higher educational attainment, greater engagement in moderate physical activity with less sedentary behavior, and a reduced use of β-blockers (Table 3).

**Table 3:** Baseline questionnaire data by quintile of urinary 6-sulfatoxymelatonin-to-creatinine ratio (aMT6s/Cr)

		aMT6s/Cr quintiles				
Variable (χ² *P* value), % missing	Category	Q1	Q2	Q3	Q4	Q5
Sleep duration (0.8), 11%	5 hours or less	61 (7.8%)	56 (7.0%)	58 (7.2%)	50 (6.2%)	59 (7.4%)
	6 hours	199 (25.5%)	194 (24.2%)	180 (22.4%)	191 (23.9%)	183 (22.9%)
	7 hours	329 (42.2%)	344 (42.9%)	348 (43.2%)	327 (40.9%)	330 (41.4%)
	≥ 8 hours	190 (23.9%)	208 (26.0%)	219 (27.1%)	232 (29.0%)	226 (28.2%)
Sleep disruption (0.1), 11%	Never	210 (27.1%)	209 (26.1%)	243 (30.3%)	234 (29.3%)	245 (30.7%)
	< 3 times/week	315 (40.6%)	336 (41.9%)	335 (41.8%)	336 (42.1%)	346 (43.4%)
	≥ 3 times/week	250 (32.2%)	257 (32.0%)	223 (27.9%)	228 (28.6%)	207 (26.0%)
Alcohol use (0.2), 7%	Never	69 (8.3%)	54 (6.5%)	57 (6.8%)	56 (6.7%)	58 (7%)
	≤1 per week	488 (58.9%)	528 (63.6%)	542 (64.8%)	520 (62.1%)	538 (64.8%)
	≥2 per week	272 (32.8%)	249 (29.9%)	237 (28.3%)	261 (31.2%)	235 (28.3%)
Smoking (0.001), 7%	Current	133 (16.0%)	137 (16.4%)	118 (14.1%)	131 (15.7%)	114 (13.7%)
	Former	249 (30%)	214 (25.7%)	204 (24.4%)	222 (26.6%)	200 (24.1%)
	Never	449 (54%)	483 (57.9%)	514 (61.5%)	483 (57.8%)	517 (62.2%)
Education level (<0.001), 7%	<9 years	78 (9.4%)	49 (5.9%)	51 (6.1%)	45 (5.4%)	31 (3.7%)
	Secondary/Vocational	491 (59.2%)	468 (56.4%)	459 (55%)	463 (55.4%)	485 (58.4%)
	University	261 (31.4%)	313 (37.7%)	324 (38.8%)	328 (39.2%)	314 (37.8%)
Physical activity (0.03), 7%	Sedentary	92 (11.1%)	65 (7.8%)	61 (7.3%)	79 (9.5%)	70 (8.5%)
	Moderate	545 (65.8%)	546 (65.7%)	561 (67.2%)	548 (65.7%)	569 (68.7%)
	Regular	191 (23.1%)	220 (26.5%)	213 (25.5%)	207 (24.8%)	189 (22.8%)
ß-blocker (<0.001), 7%	No	777 (93.5%)	803 (96.3%)	812 (97%)	817 (97.6%)	811 (97.5%)
	Yes	54 (6.5%)	31 (3.7%)	25 (3%)	20 (2.4%)	21 (2.5%)

Values are presented for individuals without type 2 diabetes at baseline. The full version of this table is available in a public repositor (30).

Data are presented as frequencies (%). *P* values are derived from χ² tests.

**Figure 2:**
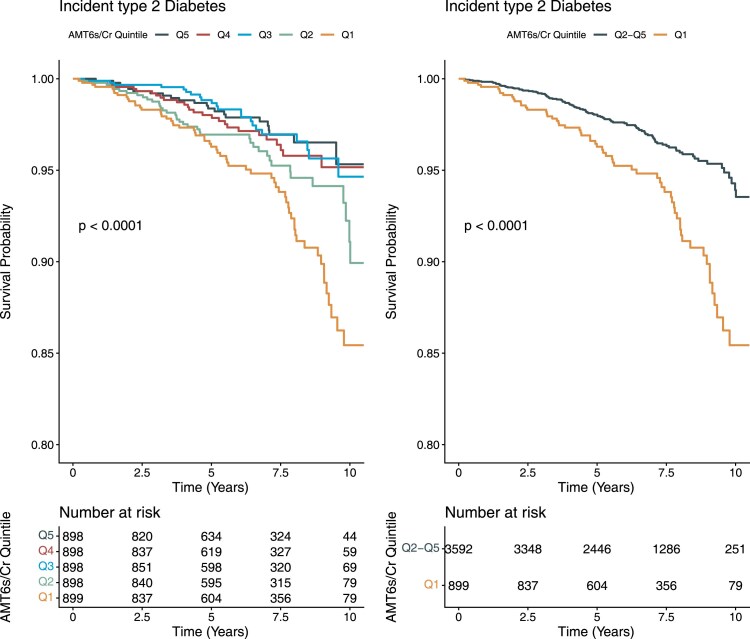
Kaplan-Meier curves of cumulative incidence of type 2 diabetes (T2D) by baseline melatonin secretion among 4491 participants. Left: all quintiles of aMT6s/Cr (Q1-Q5). Right: Q1 (lowest secretion) vs Q2-Q5 combined. Log-rank *P* values are shown. Abbreviations: aMT6s/Cr, urinary 6-sulfatoxymelatonin-to-creatinine ratio.

**Figure 3:**
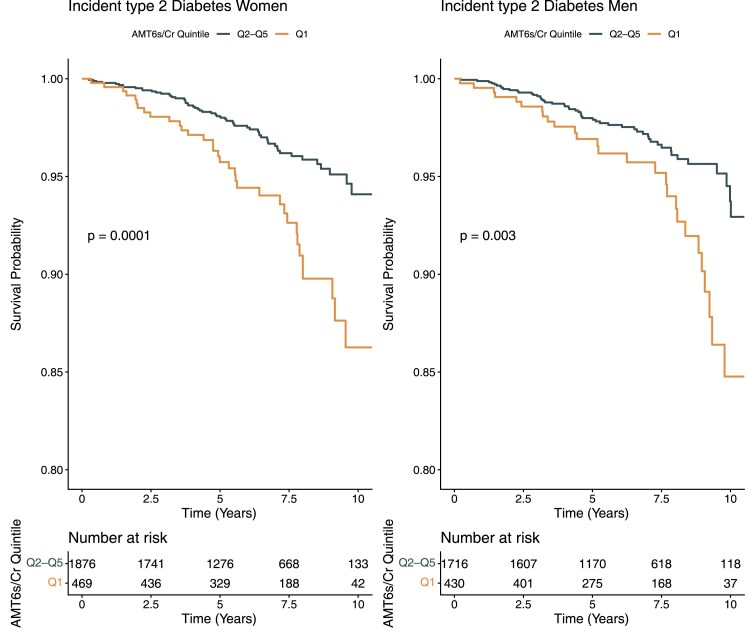
Sex-stratified Kaplan-Meier curves comparing Q1 vs Q2-Q5 of aMT6s/Cr. Left: women (n = 2345; 92 events). Right: men (n = 2146; 79 events). Log-rank *P* values are shown. Abbreviations: aMT6s/Cr, urinary 6-sulfatoxymelatonin-to-creatinine ratio.

During a median 6.5 years of follow-up, n = 171 participants developed T2D. The total follow-up time was 28 815 person-years, and the T2D incidence rate was 5.93 per 1000 person-years. Baseline aMT6s/Cr was inversely associated with incident T2D (Fig. 2). In sex-stratified analyses, aMT6s/Cr Q1 was associated with increased T2D incidence in both men and women (Fig. 3).

As shown in Table 4, participants in the lowest aMT6s/Cr quintile (Q1) exhibited a higher incidence of T2D compared to those in Q2-Q5 (hazard ratio [HR] 1.85, 95% CI 1.35-2.54).

**Table 4:** Association of urinary 6-sulfatoxymelatonin-to-creatinine ratio (aMT6s/Cr) with incident type 2 diabetes

	Hazard ratio (95% CI)	*P* value
Model 1	1.85 (1.35-2.54)	<.001
Model 2	1.50 (1.09-2.07)	.01
Model 3	1.51 (1.09-2.09)	.01
Model 4	1.54 (1.11-2.13)	.01

Hazard ratios (HRs) and 95% CIs estimated from Cox proportional hazards regression models comparing Q1 vs Q2-Q5 of aMT6s/Cr in 4491 participants (171 incident cases).

Model 1: Age. Model 2: Model 1 + BMI. Model 3: Model 2 + systolic BP, triglycerides, LDL-C, HDL-C, glucose, plasma creatinine, education, smoking, physical activity, β-blocker use. Model 4: Model 3 + sleep disruption and sleep duration.

Abbreviations: aMT6s/Cr, urinary 6-sulfatoxymelatonin-to-creatinine ratio; BP, blood pressure; HDL-C, high-density lipoprotein cholesterol; HR, hazard ratio; LDL-C, low-density lipoprotein cholesterol.

After adjustment for age and BMI, the association remained but was attenuated. In Model 3 and Model 4, participants in Q1 continued to demonstrate a higher risk for T2D compared to Q2-Q5.

In additional analyses with aMT6s/Cr as a sex-specific log-transformed and z-scored continuous variable, there was an inverse association between aMT6s/Cr and T2D (Model 1: HR 0.77, CI 0.67-0.88, *P* < .001; Model 2: HR 0.85, CI 0.74-0.98, *P* = .03; Model 3: HR 0.84, CI 0.73-0.97, *P* = .02 and Model 4: HR 0.83, CI 0.72-0.96, *P* = .01).

The proportional hazards assumption was satisfied for all reported Cox regression models and their respective covariables. Interaction testing showed no evidence that age, BMI, or sex modified the association between aMT6s/Cr and incident T2D (all *P* for interaction 0.4-1), irrespective of whether aMT6s/Cr was modeled as quintiles or standardized log-transformed values. In a sensitivity analysis excluding individuals (n = 320) who had missing data on melatonin supplementation from the Cox regression analysis, our results were unchanged (Model 3 Q1 vs Q2-Q5: HR 1.66, CI 1.18-2.33).

## Discussion

### Main findings

In this prospective cohort of adult men and women, lower nocturnal melatonin secretion was associated with a higher incidence of T2D, independent of traditional risk factors.

### Comparison with previous studies

Our results extend previous findings from the Nurses' Health Study, which linked low nocturnal melatonin secretion to incident T2D in older women (14) by demonstrating a similar association in a mixed-sex, prospective cohort with participants of varied age across a broader adult age range. Despite sex differences in absolute aMT6s/Cr levels, the relation with T2D risk was consistent in both men and women. The similarity of estimates before and after adjusting for sleep characteristics indicates that sleep did not substantially mediate or distort the observed association.

### Contextual evidence

Genetic studies indicate that increased melatonin receptor signaling impairs insulin secretion (4, 6) whereas observational data, including our results, associate low nocturnal melatonin secretion with increased T2D risk (14). This apparent contradiction highlights the possibility that melatonin may have a context-dependent role, where both insufficient secretion and excessive receptor activity can disrupt glucose regulation. Notably, much of the experimental evidence for melatonin's inhibitory effect on insulin secretion derives from nocturnal rodent models, in which melatonin peaks during their active feeding phase, whereas in humans the nocturnal rise coincides with rest and fasting, complicating direct translation of these findings (1, 12).

In humans, physiological melatonin secretion in a fasted state at night may support metabolic health by promoting β-cell survival, exerting antioxidant effects, and enhancing insulin sensitivity (1, 25, 26). In contrast, elevated melatonin during meals can impair glucose tolerance, particularly in *MTNR1B* risk-allele carriers (11, 27). We thus hypothesize that appropriately timed melatonin supplementation aligned with circadian rhythms may improve metabolic outcomes, although randomized trial findings have been inconsistent (28). Our current findings, together with previous study results, underscore the need to clarify the conditions during which melatonin supports or impairs glucose regulation.

### Strengths and limitations

The strengths of our study include the prospective design, the large sample of men and women of varying age, and near-complete follow-up through regional and national registers. The association between aMT6s/Cr and incident T2D was consistent across categorical and continuous specifications, supporting robustness of the findings. Several limitations should also be acknowledged. First, reliance on a single morning urine sample to approximate nocturnal melatonin secretion does not capture day-to-day variability and may introduce exposure misclassification. Because the direction and magnitude of such measurement error are uncertain, its influence on the observed associations may be either attenuating or distorting. Second, although we included a wide range of covariates, residual confounding remains possible. We did not have data on evening light exposure, chronotype, shift-work history, and sleep disorders, including obstructive sleep apnea, which are key circadian and behavioral determinants with biological relevance to both melatonin secretion (1-3, 13) and metabolic risk (2, 3, 11, 13). Their absence limits our ability to fully address confounding from these domains. Third, genetic information related to metabolism or T2D susceptibility was not available, limiting our ability to address potential genetic confounding. Finally, the predominance of participants of European descent may reduce generalizability. Future studies incorporating repeated aMT6s/Cr assessments across several nights, together with richer circadian, sleep, and genetic phenotyping, are warranted.

### Clinical implications

Our findings suggest that low nocturnal melatonin secretion may serve as an early biomarker of T2D risk, complementing established metabolic and lifestyle predictors. The association persisted after adjustment for sleep patterns and metabolic risk factors, underscoring the role of circadian biology in T2D development. As melatonin use in Sweden has increased substantially in recent years (29), it is important to clarify how exogenous supplementation interacts with endogenous secretion and influences T2D risk. Future studies should assess whether circadian aligned interventions through appropriately timed melatonin administration, light exposure, sleep timing, or meal timing, can reduce T2D risk, particularly in genetically susceptible groups. A recent meta-analysis of randomized trials reported heterogeneous effects of supplementation on glucose metabolism and diabetes risk (28), highlighting the need for long-term studies on safety and efficacy.

## Conclusion

Low nocturnal melatonin secretion was independently associated with higher risk of T2D in this prospective cohort of men and women of varied age, emphasizing circadian regulation as a determinant of metabolic health and supporting evaluation of melatonin as a biomarker and potential preventive target.

## Data Availability

The data underlying this article were collected within the Malmö Offspring Study. Due to Swedish and EU data protection regulations, individual-level data cannot be made publicly available. Researchers may apply for data access through the Malmö Offspring Study steering committee. Information about access procedures is available at https://www.malmo-kohorter.lu.se.

## Abbreviations

aMT6s/Cr: 6-sulfatoxymelatonin-to-creatinine ratio
BMI: body mass index
HR: hazard ratio
MOS: Malmö Offspring Study
Q1: quintile 1
T2D: type 2 diabetes
